# Racial-ethnic differences in prevalence of social determinants of health and social risks among middle-aged and older adults in a Northern California health plan

**DOI:** 10.1371/journal.pone.0240822

**Published:** 2020-11-04

**Authors:** Nancy P. Gordon, Matthew P. Banegas, Reginald D. Tucker-Seeley

**Affiliations:** 1 Kaiser Permanente Division of Research, Oakland, California, United States of America; 2 Kaiser Permanente Center for Health Research, Portland, Oregon, United States of America; 3 Leonard Davis School of Gerontology, University of Southern California, Los Angeles, California, United States of America; Yale School of Medicine, UNITED STATES

## Abstract

**Background:**

Social determinants of health (SDoHs) and social risks (SRs) have been associated with adverse health and healthcare utilization and racial/ethnic disparities. However, there is limited information about the prevalence of SRs in non-“safety net” adult populations and how SRs differ by race/ethnicity, age, education, and income.

**Methods:**

We analyzed weighted survey data for 16,247 White, 1861 Black, 2895 Latino, 1554 Filipino, and 1289 Chinese adults aged 35 to 79 who responded to the 2011 or 2014/2015 Kaiser Permanente Northern California Member Health Survey. We compared age-standardized prevalence estimates of SDoHs (education, household income, marital status) and SRs (financial worry, cost-related reduced medication use and fruit/vegetable consumption, chronic stress, harassment/discrimination, health-related beliefs) across racial/ethnic groups for ages 35 to 64 and 65 to 79.

**Results:**

SDoHs and SRs differed by race/ethnicity and age group, and SRs differed by levels of education and income. In both age groups, Blacks, Latinos, and Filipinos were more likely than Whites to be in the lower income category and be worried about their financial situation. Compared to Whites, cost-related reduced medication use was higher among Blacks, and cost-related reduced fruit/vegetable consumption was higher among Blacks and Latinos. Younger adults were more likely than older adults to experience chronic stress and financial worry. Racial/ethnic disparities in income were observed within similar levels of education. Differences in prevalence of SRs by levels of education and income were wider within than across racial/ethnic groups.

**Conclusions:**

In this non-“safety net” adult health plan population, Blacks, Latinos, and Filipinos had a higher prevalence of social risks than Whites and Chinese, and prevalence of social risks differed by age group. Our results support the assessment and EHR documentation of SDoHs and social risks and use of this information to understand and address drivers of racial/ethnic disparities in health and healthcare use.

## Introduction

In recent years, medical and public health organizations have shown an increasing interest in estimating the prevalence of different social determinants of health (SDoHs), social/financial risks, and actual social/economic needs in the populations they serve and understanding the impact of these social factors on population health and healthcare utilization [1–7]. Screening for and employing cost-effective methods of addressing both medical-related and non-medical social and financial needs through individual- and population-level interventions is being seen as a necessary next step to achieving the Triple Aim of better health, improved health care delivery, and reduced healthcare costs [8–11]. A growing body of research also suggests that SDoHs, especially educational attainment and income, and social/environmental risk factors closely associated with these SDOHs, may be significant drivers of observed patterns of racial/ethnic disparities in health and health care utilization [12–17]. Accordingly, it is important to characterize racial/ethnic differences in SDoHs and social/financial risks as these may have implications for improving healthcare delivery and health outcomes.

Alderwick and Gottlieb (2019) propose a distinction between the terms SDoHs, social risk factors (SRs), and social needs [18]. SDoHs such as educational attainment, income, marital status, healthcare access, and characteristics of the work and neighborhood environment, are factors that affect everyone and that can positively or adversely affect health, sense of well-being, and healthcare use at the individual and population level. Previous research suggests that adults with high educational attainment and high income are more likely than those with low educational attainment and low income to engage in health protective lifestyle behaviors and to avoid adverse health behaviors, to access recommended preventive medical and dental care, and to have better health and longer life expectancy [19–23]. Similarly, being married or in a committed relationship can impact health and well-being depending on whether the relationship provides emotional and instrumental social support and contributes to financial well-being or is a source of psychological or physical abuse and financial stress. SRs are factors that can lead to individual-level circumstances not conducive to good health. These include the adverse effects of SDoHs (low educational attainment, low income, not being married or in a committed relationship that contributes instrumental and social support) and circumstances that may result from them, including low health literacy, having no job or a low paying job, financial strain, chronic stress, and a low level of social support. Social needs are those SRs that individuals or their healthcare or social service providers determine are adversely affecting the individual’s or their family’s health and well-being, such as being homeless, socially isolated or lonely, food or medicine insecure, or transportation-challenged.

There is little information about the prevalence of SRs in non-“safety net” (not extremely low income) adult populations and how SRs vary by sociodemographic (i.e., age, sex, race/ethnicity) and SDoH factors (income level and educational attainment). There is also a knowledge gap regarding whether, in this type of adult population, the prevalence of SRs within and across racial/ethnic groups varies by age group, income level, and educational attainment. To address these gaps, we used data from general health surveys conducted in 2011 and 2014/2015 at Kaiser Permanente Northern California to describe and compare the prevalence of SDoHs and SRs among middle-aged and older adult non-Hispanic White, Black, Latino, Filipino, and Chinese adult members in this health plan member population. We also examined how two SDoHs, educational attainment and income, are associated with SRs within and across racial/ethnic groups.

## Materials and methods

### Setting

Kaiser Permanente Northern California (KPNC) provides integrated primary and specialty health care to a racially/ethnically and sociodemographically diverse health plan membership that includes over 3.2 million adults who mostly reside in the San Francisco Bay and Greater Bay Area, Sacramento area, Silicon Valley, and Central Valley. A very small percentage of KPNC adult members (ages 25 to 64: 2% in 2014 and 4% in 2015; ages 65 to 85: 0.5% in both years) would be considered “safety net”, i.e., extremely low income individuals covered through the Medicaid public health insurance program. The KPNC adult membership was previously shown to be very similar to the non-Medicaid covered insured population of Northern California in sociodemographic and health characteristics [24].

### Data source

The KPNC adult Member Health Survey (MHS) is a self-administered (mailed print or online questionnaire) survey conducted with an age-gender-geographically stratified random sample of health plan members aged 20 years and over who were members at the time of the survey (Spring of a survey year) and during at least the fourth quarter of the preceding year. The MHS is only conducted in English and oversamples older adults. The survey collects information on a wide range of variables related to health and health care use, including sociodemographic characteristics (e.g., race/ethnicity, educational attainment, household income, employment status, marital status, sexual orientation), health status, health-related behaviors and psychosocial risks, and use of digital communication technologies. More information about the survey is found in an earlier publication [25]. The survey response rate was approximately 35% for the 35 to 64 age group in both the 2011 and 2014/2015 survey cycles and 69% and 64% for the 65 to 79 age group in the 2011 and 2014/15 cycles, respectively. The MHS and analyses undertaken for this study were approved by the KPNC Institutional Review Board.

### Survey sample

For this study, we used pooled survey data obtained from the 2011 and 2014/2015 MHS cycles for 23,846 adults aged 35 to 79 who were classified based on self-report as non-Hispanic White (White, n = 16,247), Black (n = 1861), Hispanic/Latino (n = 2895), Filipino (n = 1554), or Chinese (n = 1289), the KPNC health plan’s five largest adult racial/ethnic groups. Our decision to analyze data for Filipinos and Chinese rather than for Asians was based on previous analyses of MHS data that found significant differences in health characteristics between these two Asian ethnic groups. Because preliminary analyses identified substantial age cohort differences in SDoHs, we analyzed data for two age groups: 35 to 64 years (n = 8595 Whites, 1151 Blacks, 2001 Latinos, 1015 Filipinos, and 918 Chinese) and 65 to 79 years (n = 7652 Whites, 710 Blacks, 894 Latinos, 539 Filipinos, and 371 Chinese). The sex distribution was approximately equal across these age groups. None of these MHS respondents were Medicaid enrollees, but most of the older group was covered by Medicare, the U.S. government-funded health insurance program available to U.S. citizens aged 65 and over and other special populations.

### Study variables

This study examined data on three SDoHs (educational attainment, household income, and marital status) and seven SRs (financial worry, reduced medication use due to cost, reduced fruit and vegetable consumption due to cost, experience of harassment/discrimination in the past 12 months, chronic high level of stress, and beliefs that health habits/lifestyle and stress/emotional troubles can have a large effect on their health) ascertained by the survey. Educational attainment was examined using four levels: non-high school graduate, high school graduate, some college, and college graduate (bachelor’s degree or higher). Household income was examined using four categories: very low income (< $25,000); lower income (< $35,000, the cut-point for the lower tertile of household income for Northern California adults in 2015 for ages 35 to 79 and for both age groups); ≥ $65,000 (the approximate median household income for all adults aged 35 to 79 in Northern California in 2015 [26]), and for ages 35 to 64, higher income (≥ $100,000, the upper tertile of household income for that age group in Northern California in 2015). Because the upper tertile for household income for ages 65 to 79 fell between two possible income category cut-points (≥ $65,000 was the upper 37^th^ percentile and ≥ $80,000 was the upper 28^th^ percentile), for the older adult group we also report percentages with a household income of ≥ $80,000. Marital status was categorized as married or not married. The SRs were all dichotomized as present or absent. The exact wording of the questions and responses used to create the variables is found in S1 Appendix.

### Statistical analysis

Data were analyzed using SAS version 9.4 (SAS Institute, Cary, IN, 2013) procedures for data obtained from complex survey designs [27]. All analyses used data weighted to reflect the age-sex composition of White, Black, Latino, Filipino, and Chinese adults ages 35 to 79 in the KPNC membership. To facilitate direct comparison of prevalence of SDoH and SRs across racial/ethnic groups and within levels of income and education, we used the Proc Surveyreg procedure recommended by the Centers for Disease Control and Prevention to age-sex standardize estimates to the 2010 US Census [28], using separate standardization models for the 35 to 64 and 65 to 79 age groups. The prevalence statistics reported in the tables and figures are all age-standardized. A second step in the Proc Surveyreg models assessed the statistical significance of differences in SDoHs and SRs both between racial/ethnic groups and within racial/ethnic groups, based on high and low education and income strata. For comparisons involving income, we used<$35,000 as the lower income category for both age groups and for higher income, ≥ $100,000 for ages 35 to 64 and ≥$65,000 for ages 65 to 79. For comparisons by educational attainment, we used high school graduate versus college graduate. All racial/ethnic group comparisons used Whites as the reference group except when the comparison was restricted to Filipino versus Chinese adults. Multivariable logistic regression models were used to test for differences in SDoHs and SRs by race/ethnicity, income, and education, controlling for age group and sex in models that assessed differences between the younger and older age groups within the same racial/ethnic group or same education or income strata. We did not adjust for multiple comparisons but report the results of all statistical tests.

## Results

Table 1 shows that in the 35 to 64 age group, Blacks and Latinos were less likely than Whites to have low educational attainment (no formal education beyond high school), with Latinos having higher percentages with very low and low educational attainment than Blacks. Educational attainment among Chinese was higher than that of Whites, and among Filipinos, comparable to Whites but lower than Chinese. Across all groups except the Chinese group, men were more likely than women to have no formal education beyond high school. Higher percentages of Blacks, Latinos, and Filipinos than Whites were in the lower household income category. Across all racial/ethnic groups, women were less likely than men to be in the higher income category. Blacks were significantly less likely than all of the other racial/ethnic groups to be married, while Filipinos and Chinese were more likely than the other groups to be married. With the exception of Filipinas, women were less likely than men to be married.

**Table 1:** Racial/Ethnic group differences in social determinants of health, men and women aged 35–64.

Characteristic	White % (95% CI)	Black % (95% CI)	Latino % (95% CI)	Filipino % (95% CI)	Chinese % (95% CI)
**Social determinants**					
**Education**					
** ≤ High school graduate**	17.0% ^a^(16.0–18.1)	23.6%^a^^,^^b^ (20.9–26.4)	34.7 ^a^^,^^b^ (32.5–37.0)	14.6 ^a^^,^^c^ (12.2–17.0)	8.7^d^ (6.7–10.8)
** Non-high school graduate**	1.5 (1.1–1.8)	1.5 (0.7–2.2)	6.9 ^b^ (5.7–8.0)	1.8 (1.0–2.7)	1.8 (0.9–2.6)
** College Graduate**	51.3 (49.9–52.6)	32.3 ^d^ (29.3–35.3)	28.1 ^d^ (25.9–30.4)	53.0^e^^,^^f^ (49.7–56.2) ^c^	69.9 ^b^ (66.8–73.1)
**Household income**					
** < $35,000**	9.7 (8.8–10.5)	21.5 ^b^ (19.0–24.0)	14.9 ^a^^,^^b^ (13.2–16.6)	12.8 ^b^^,^^c^ (10.6–14.9)	8.4 (6.6–10.3)
** < $25,000**	6.1 (5.4–6.8)	14.5 ^b^ (12.3–16.8)	7.0 (5.7–8.2)	5.9 (4.4–7.5)	4.8 (3.3–6.3)
** ≥ $65,000**	70.8 ^a^ (69.6–72.1)	47.9 ^a^^,^^d^ (44.7–51.1)	54.9 ^a^^,^^d^ (52.5–57.4)	59.6 ^d^^,^^e^ 56.4–62.9	71.9 ^a^ (68.8–74.9)
** ≥ $100,000**	42.3 ^a^ (40.9–43.7)	22.3 ^a^^,^^d^ (19.4–25.1)	25.3 ^d^ (23.0–27.6)	33.5 ^a^^,^^d^^,^^e^ (30.3–36.8)	48.4^a^^,^^b^ (45.0–51.9)
**Marital status (% married)**	69.9 ^a^ (68.6–71.2)	50.5 ^a^^,^^d^ (47.3–53.6)	69.9 ^a^ (67.7–72.2)	75.1 ^b^ (72.2–77.9)	77.7 ^a^^,^^b^ (74.8–80.6)
**Social risks**					
**Worried a great deal about financial situation in past year**	29.6 ^f^ (28.3–30.8)	40.2 ^b^^,^^f^ (37.1–43.3)	34.7 ^b^^,^^f^ (32.3–37.0)	33.8 ^b^^,^^c^ (30.7–36.9)	21.4^d^ (18.5–24.2)
**Used less medication than prescribed due to cost**	7.5 (6.7–8.2)	12.3 ^b^ (10.2–14.3)	9.2 ^b^ (7.8–10.6)	7.6 (3.8–9.3)	7.0 (5.4–8.7)
**Ate less fruits and vegetables than wanted due to cost** ^g^	8.0 ^f^ (7.1–8.9)	16.3 ^b^ (13.1–19.6)	12.7 ^b^^,^^f^ (10.8–14.7)	6.8 (4.4–9.2)	4.9 ^d^ (2.9–7.0)
**Experienced harassment or discrimination in past year**	4.9 (4.3–5.5)	12.9 ^b^ (10.8–15.0)	8.1 ^b^ (6.7–9.5)	5.9 (4.3–7.5)	5.5 (3.9–7.2)
**Has chronic high level of stress**	20.0 ^f^ (18.8–21.1)	21.5 ^f^ (18.9–24.2)	17.2 ^f^ (15.4–19.1)	6.1 ^d^^,^^f^ (3.6–8.5)	7.4 ^d^^,^^f^ (5.0–9.9)
**Health-related beliefs:**					
** Health habits/lifestyle have a large impact on health** ^g^	92.5 (91.6–93.4)	87.2 ^d^ (84.3–90.2)	87.7 ^d^^,^^f^ (85.7–89.6)	75.7 ^d^^,^^e^ (71.7–79.8)	84.9 ^d^ (81.5–88.2)
** Stress/emotional troubles have a large impact on health** ^g^	89.9 (88.9–90.9)	86.6 ^d^ (83.6–89.6)	83.4 ^d^^,^^f^ (81.1–85.6)	71.5 ^d^^,^^e^ (67.3–75.8)	82.9 ^d^ (79.3–86.4)

^a^ Significantly (p<.05) higher among men than women within this race/ethnic group.

^b^ Significantly (p<.05) higher than non-Hispanic Whites.

^c^ Filipinos significantly (p<.05) higher than Chinese.

^d^ Significantly (p<.05) lower than non-Hispanic Whites.

^e^ Filipinos significantly (p<.05) lower than Chinese.

^f^ Significantly (p<.05) higher among women than men within this race/ethnic group.

^g^ Based on survey data from the 2014/2015 cycle only.

Blacks, Latinos, and Filipinos were more likely than Whites, while Chinese were less likely than both Whites and Filipinos, to indicate being worried about their financial situation. Among Whites, Blacks, and Latinos, women were more likely than men to be worried about their financial situation. Blacks were more likely than all other racial/ethnic groups and Latinos were more likely than Whites and Chinese to report that during the previous year they had taken less medication than prescribed due to cost. Blacks and Latinos were also more likely than the other groups to report that they had eaten less fruits and vegetables than they wanted due to cost.

Blacks were more likely than all other racial/ethnic groups, and Latinos were more likely than Whites, Filipinos, and Chinese to report having experienced harassment or discrimination in the past year. Filipinos and Chinese were less likely than Whites, Blacks, and Latinos to experience high levels of stress. Across all racial/ethnic groups, women were more likely than men to report chronic high levels of stress. Significantly higher percentages of Whites compared with the other four racial/ethnic groups believed that health habits/lifestyle and stress/emotional troubles could have a large effect on their health, with Filipinos less likely to hold these beliefs than all of the other racial/ethnic groups.

Table 2 shows that in the older age group, compared to Whites, higher percentages of Blacks and Latinos and lower percentages of Chinese had no formal education beyond high school. Blacks, Latinos, and Filipinos had higher percentages with very low educational attainment, with Latinos having the highest percentages with low and very low educational attainment. Among Whites, Latinos, and Chinese, women were more likely than men to have no formal education beyond high school. Blacks, Latinos, and Filipinos were more likely than Whites and Chinese to be in the very low and low income categories and less likely than both Whites and Chinese to be in the above median and higher income categories. Across all five racial/ethnic groups, women were less likely than men to be in the higher income categories. As in the younger group, Blacks were less likely than the other racial/ethnic groups to be married and Filipino and Chinese adults were more likely to be married, with women less likely than men to be married in all five racial/ethnic groups.

**Table 2:** Racial/Ethnic group differences in social determinants of health, men and women aged 65–79.

Characteristic	White % (95% CI)	Black % (95% CI)	Latino % (95% CI)	Filipino % (95% CI)	Chinese % (95% CI)
**Social determinants**					
**Education**					
** ≤ High school graduate**	27.0 ^a^ (25.5–28.4)	37.5 ^b^ (33.5–41.5)	51.5 ^a^^,^^b^ (47.8–55.2)	24.9 (21.1–28.7)	21.7 ^a^^,^^c^ (17.3–26.1)
** Non-high school graduate**	4.9 (4.3–5.6)	8.2 ^b^ (6.2–10.2)	16.7 ^b^ (14.2–19.2)	9.1 ^b^^,^^d^ (6.7–11.5)	3.9 (2.1–5.6)
** College Graduate**	40.5 ^f^ (39.0–42.1)	26.3 ^c^ (22.4–30.1)	15.6 ^c^^,^^f^ (12.7–18.5)	53.2 ^b^ (48.6–57.8)	52.9 ^b^^,^^f^ (47.2–58.5)
**Household income**					
** < $35,000**	25.8 ^a^ (24.4–27.2)	34.4 ^a^^,^^b^ (30.7–38.1)	38.4 ^a^^,^^b^ (34.9–41.8)	40.0 ^b^^,^^d^ (35.6–44.3)	23.4 (18.8–27.9)
** < $25,000**	16.9 ^a^ (15.6–18.1)	25.6 ^a^^,^^b^ (22.1–29.1)	24.9 ^a^^,^^b^ (21.9–28.0)	29.8 ^b^^,^^d^ (25.8–33.8)	16.1 (12.1–20.2)
** ≥ $65,000**	40.8 ^f^ (39.2–42.5)	30.8 ^c^^,^^f^ (26.6–35.0)	25.1 ^c^^,^^f^ (21.6–28.7)	24.8 ^c^^,^^e^^,^^f^ (20.3–29.3)	47.2 ^b^^,^^f^ (41.2–53.1)
** ≥ $80,000**	28.4 ^f^ (26.9–30.0)	20.5^,^^f^ (16.7–24.3)	13.9 ^c^^,^^f^ (10.8–16.9)	15.2 ^c^^,^^e^^,^^f^ (11.3–19.2)	34.4 ^b^^,^^f^ (28.5–40.2)
**Marital status (% married)**	63.3 ^f^ (61.8–64.8)	50.6 ^c^^,^^f^ (46.7–54.5)	60.9 ^f^ (57.3–64.5)	72.3 ^b^^,^^f^ (68.5–76.0)	76.4 ^b^^,^^f^ (71.7–81.2)
**Social risks**					
**Worried a great deal about financial situation in past year**	14.1 ^a^ (13.0–15.3)	20.0 ^b^ (16.6–23.4)	18.8 ^b^ (15.7–21.1)	21.8 ^b^^,^^d^ (17.9–25.7)	10.1 ^c^ (6.6–13.7)
**Used less medication than prescribed due to cost**	5.6 (4.8–6.3)	8.0 ^b^ (5.7–10.4)	6.0 (4.2–7.7)	6.6 (4.3–9.0)	4.2 (1.7–6.6)
**Ate less fruits and vegetables than wanted due to cost** ^g^	4.1 ^a^ (3.3–4.8)	7.5 ^b^ (4.5–10.6)	9.1 ^a^^,^^b^ (6.1–12.2)	4.6 (1.9–7.3)	2.1 (0–4.5)
**Experienced harassment or discrimination in past year**	2.4 (1.9–2.9)	6.2 ^b^ (4.2–8.3)	1.8 (0.9–2.8)	2.5 (1.1–4.0)	3.5 (1.5–5.5)
**Has chronic high level of stress**	6.2 (5.4–7.0)	5.3 (3.3–7.3)	5.5 (3.6–7.4)	4.9 (2.6–7.1)	2.1 ^c^ (0.3–3.9)
**Health-related beliefs:**					
** Health habits/lifestyle have a large impact on health** ^g^	90.3 (89.3–90.1)	86.4 (82.7–90.6)	79.2 ^c^ (75.1–83.2)	59.3 ^c^^,^^e^ (53.0–65.7)	80.4 ^c^ (74.5–86.2)
** Stress/emotional troubles have a large impact on health** ^g^	82.5 ^a^ (81.2–83.8)	78.1 (73.4–82.7)	71.3 ^c^ (66.7–75.8)	48.6 ^a^^,^^c^^,^^e^ (42.2–55.0)	74.5 ^a^^,^^c^ (68.1–80.9)

^a^ Significantly (p<.05) higher among women than men within this race/ethnic group.

^b^ Significantly (p<.05) higher than non-Hispanic Whites.

^c^ Significantly (p<.05) lower than non-Hispanic Whites.

^d^ Filipinos significantly (p<.05) higher than Chinese.

^e^ Filipinos significantly (p<.05) lower than Chinese.

^f^ Significantly (p<.05) higher among men than women within this race/ethnic group.

^g^ Based on survey data from the 2014/2015 cycle only.

Blacks, Latinos, and Filipinos were more likely than Whites, while Chinese were less likely than both Whites and Filipinos, to indicate being worried about their financial situation. Blacks were more likely than Whites to report having taken less medication than prescribed due to cost during the past year, and Blacks and Latinos were more likely than Whites to say they had eaten less fruits and vegetables than they wanted due to cost. Blacks were more likely than all other racial/ethnic groups to report having experienced harassment or discrimination in the past year. Whites were more likely than the other racial/ethnic groups and Filipinos less likely than all other groups to believe that their health habits/lifestyle could greatly affect their health, and Latinos, Filipinos and Chinese were less likely than Whites to believe that stress and emotional troubles could greatly affect their health.

A comparison of the older and younger adults within racial/ethnic groups (Tables 1 and 2) showed that across all racial/ethnic groups, the older adults had higher percentages with low educational attainment (high school diploma or less) and, with the exception of Filipinos, lower percentages with a college degree. Similarly, across all racial/ethnic groups, significantly higher percentages of older adults were in the lower income (< $35,000) and very low income (< $25,000) categories. Among Whites and Latinos, older adults were significantly less likely to be married, but no age group difference in marital status was seen in the other racial/ethnic groups.

Within all racial/ethnic groups, the older adults were less likely to indicate that in the past year they had worried a great deal about their financial situation and to have experienced chronic high stress. Whites and Latinos in the older group were less likely than those in the younger group to indicate that they had eaten less fruits and vegetables and used less medication in the past year due to the cost. With the exception of Chinese adults, adults in the older group were less like to indicate having experienced incidents of harassment or discrimination in the past year. Whites, Filipinos, and Latina and Chinese women in the older group were less likely than those in the younger group to believe that their health habits/lifestyle can have a large effect on their health. Whites, Blacks, Latinos, Filipinos, and Chinese women in the older age group were less likely than those in the younger group to believe that stress and emotional troubles can have a large effect on their health.

**Fig 1:**
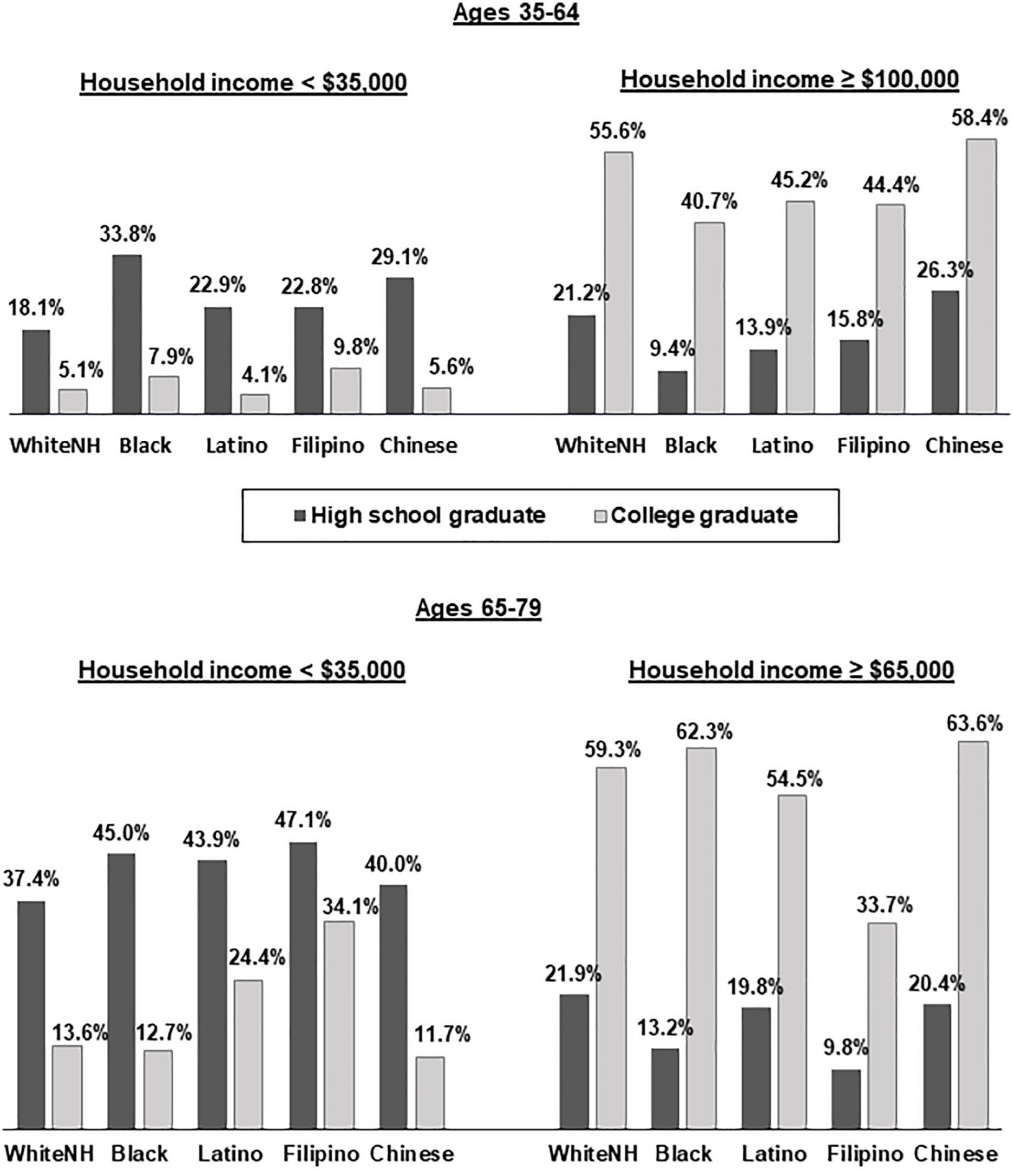
Percentages of adults in lower and higher income categories by educational attainment, race/ethnicity and age group. All percentages are age-sex standardized. All differences between lower and higher levels of education within racial/ethnic groups are statistically significant at p<.05.

Fig 1 shows the relationship of educational attainment (high school graduate versus college graduate) with prevalence of lower and higher household income levels. Among high school graduates, Blacks, Latinos, and Chinese in the younger age group and Blacks in the older age group were more likely than Whites to be in the lower income group. At that same education level, Blacks and Latinos in the younger age group and Blacks and Filipinos in the older age group were less like than Whites to be in the higher income category. Among college graduates, Blacks and Filipinos in the younger group and Latinos and Filipinos in the older group were more likely than Whites to be in the lower income category, and Blacks, Latinos, and Filipinos in the younger group and Filipinos in the older group were less likely than Whites to be in the higher income category.

**Fig 2:**
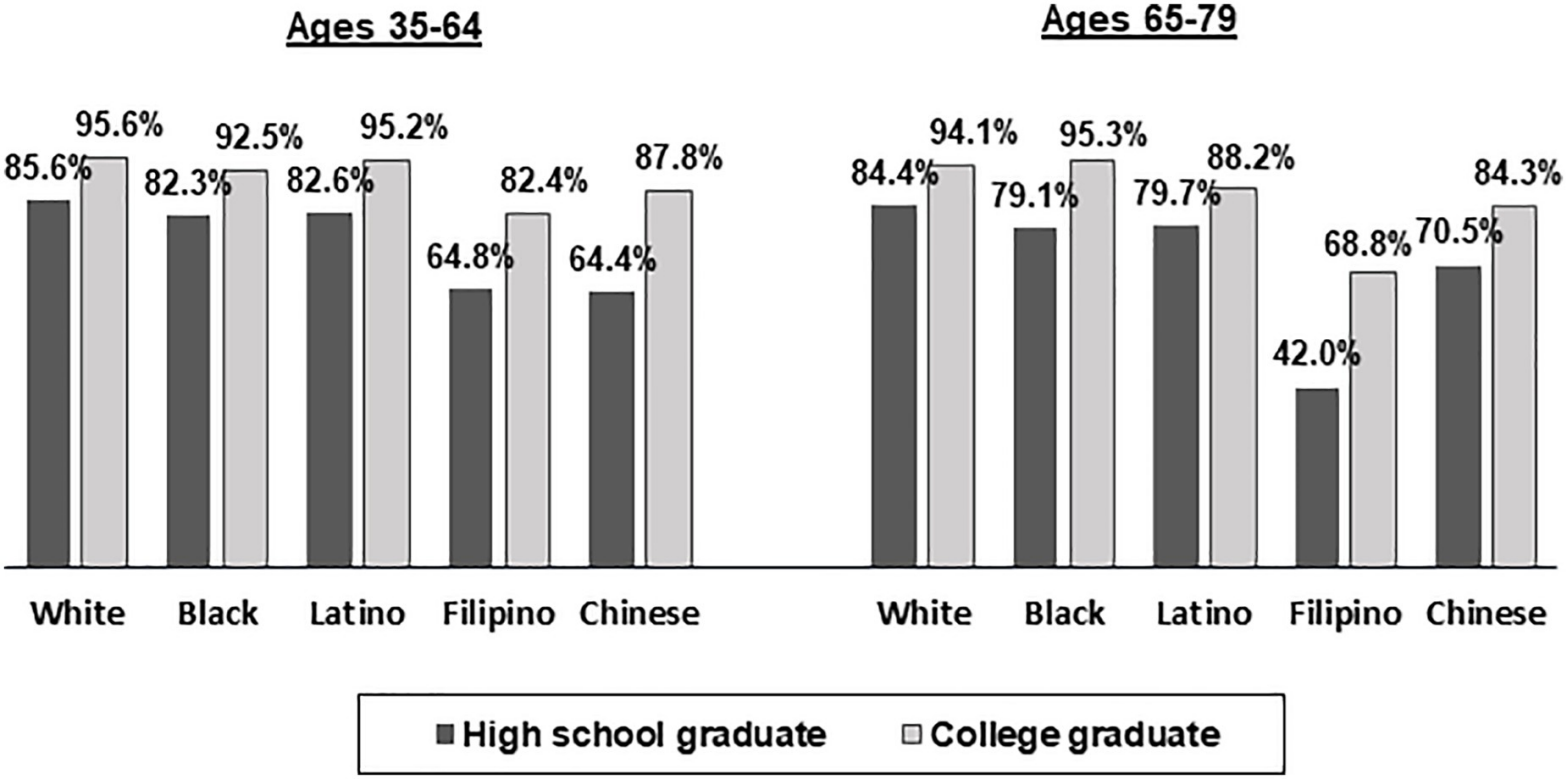
Percentages of adults at lower and high levels of education who believe that their health habits/lifestyle can have a large effect on their health, by race/ethnicity and age group. All percentages are age-sex standardized. All differences between lower and higher levels of education within racial/ethnic groups are statistically significant at p<.05.

Fig 2 shows a comparison of percentages of adult high school graduates and college graduates who believe that their health habits/lifestyle can have a large effect on their health. Across all racial/ethnic groups in both age groups, college graduates were more likely to hold this belief than high school graduates. Within education levels, Blacks and Latinos did not significantly differ from Whites, but Filipino (both age groups) and Chinese (older age group only) high school graduates and Filipino and Chinese college graduates (both age groups) were less likely to hold this belief than Whites. Black, Latino and Chinese adults in the younger age group and White, Latino, and Chinese adults in the older age group who did not complete high school were less likely than high school graduates to hold this belief.

**Fig 3:**
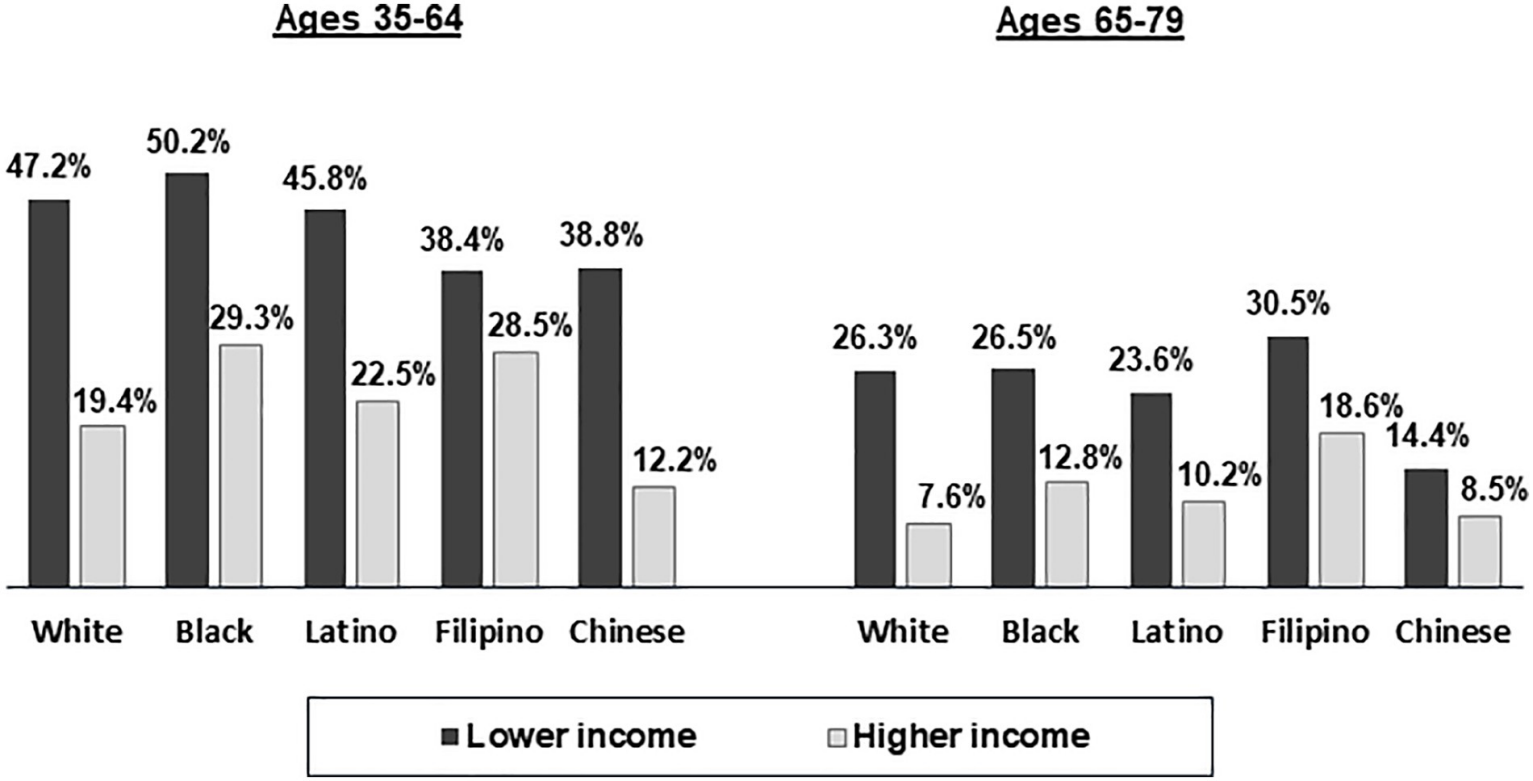
Percentages of adults in lower and higher income categories who worried a great deal about their financial situation by race/ethnicity and age group. Lower income:<$35,000 for both age groups; Higher income: ≥ $100,000 for ages 35–64 and ≥ $65,000 for ages 65–79. All percentages are age-sex standardized. All differences between lower and higher income groups within racial/ethnic groups for ages 35–64 and within White, Black, Latino, and Filipino groups for ages 65–79 are statistically significant at p<.05.

**Fig 4:**
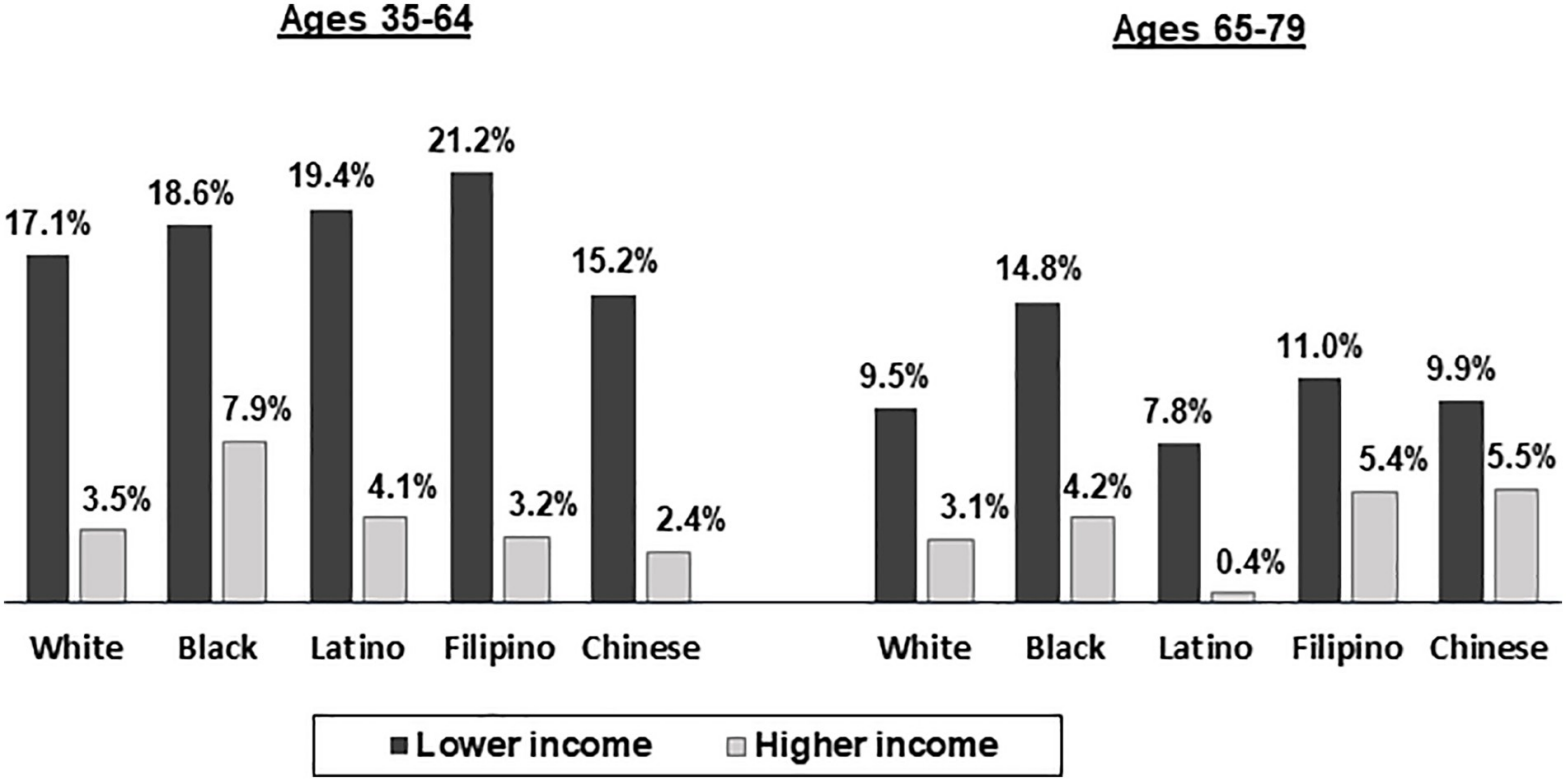
Percentages of adults in lower and higher income categories who in the past year used less medication than prescribed due to cost, by race/ethnicity and age group. Lower income:<$35,000; Higher income: ≥ $100,000 for ages 35–64 and ≥ $65,000 for ages 65–79. All percentages are age-sex standardized. All differences between lower and higher income groups within race/ethnicity for the 35–64 age group and for Whites, Blacks, and Latinos in the 65–79 age group are statistically significant at p<.05.

**Fig 5:**
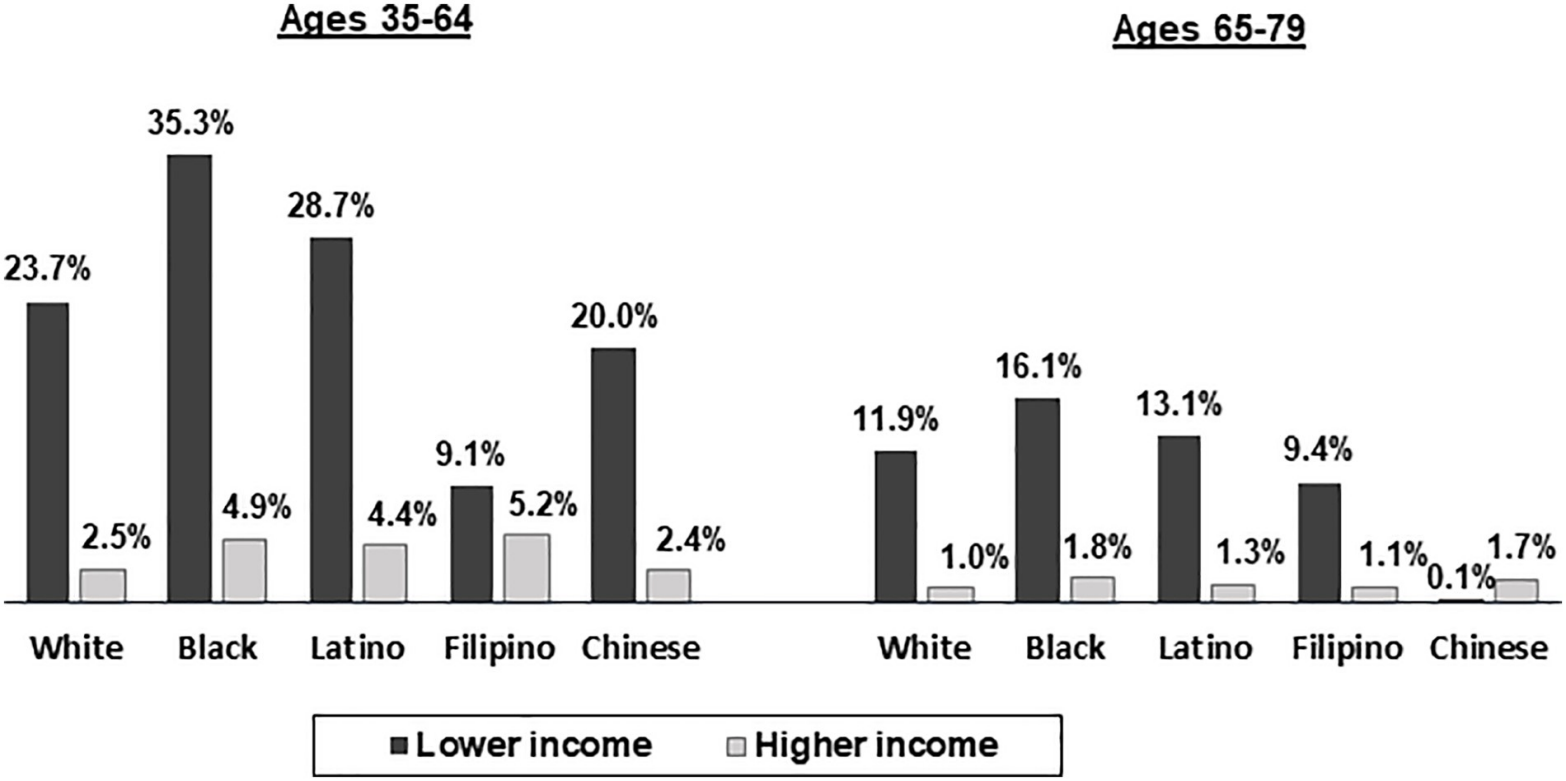
Percentages of adults in lower and higher income categories who in the past year ate less fruits and vegetables than wanted to due to cost, by race/ethnicity and age group. Lower income:<$35,000; Higher income: ≥ $100,000 for ages 35–64 and ≥ $65,000 for ages 65–79. All percentages are age-sex standardized. Differences between lower and higher income groups for Whites, Blacks, Latinos, and Chinese in the 35–64 age group and for Whites, Blacks, and Latinos in the 65–79 age group are statistically significant at p<.05.

Figs 3–5 show the associations of being in a lower versus higher income category with financial worry, under-use of prescribed medication due to cost, and reduced fruit and vegetable consumption due to cost, respectively. Across all racial/ethnic groups in the younger age group and all racial/ethnic groups except for Chinese in the older age group, substantially higher percentages of adults in the lower versus higher income category indicated being very worried about their financial situation (Fig 3). Across all racial/ethnic groups, younger adults in both the lower and higher income categories were more likely than older adults in the same income category to be very worried about their financial situation. Higher percentages of adults in the lower versus higher income category reported under-utilizing prescribed medication due to cost across all racial/ethnic groups in the 35 to 64 age group and for Whites, Blacks and Latinos in the 65 to 79 age group (Fig 4). Among lower income Whites and Latinos, younger adults were more likely than older adults to report having under-used prescription medication due to cost. In both age groups, White, Black, and Latino adults in the lower income category were significantly less likely than those in the higher income category to say that they ate less fruits and vegetables than they wanted to due to cost (Fig 5).

Finally, for the 35 to 64 age group, we examined the association of experiencing a chronic high level of stress with income, being very worried about one’s financial situation, and experiencing harassment or discrimination in the past year using logistic regression models for that simultaneously controlled for all three social factors as well as age and sex. Separate models were run for each racial/ethnic group, and all models yielded similar results. Experiencing financial worry increased the odds of chronic stress by a factor of 3 to 4 and harassment/discrimination by a factor of 2 to 4. Being in the low income category was also a significant factor, but only for Whites and Blacks. We did not examine the association of these factors with chronic stress in the older age group because the prevalence of chronic stress was so low.

## Discussion

Findings from this study of a non-“safety net” insured adult population highlight significant racial/ethnic disparities in SDoHs and SRs that likely contribute to well-documented racial/ethnic disparities in prevalence of unhealthy behaviors, lifestyle risks, and poorer health and well-being. For example, Blacks and Latinos were more likely than Whites to have low educational attainment, while Chinese were more likely than Whites to be college graduates. Blacks, Latinos, and Filipinos were significantly more likely than Whites to be in the lower income category and to be very worried about their financial situation. Blacks and Latinos were also more likely than Whites to report that they had consumed less fruits and vegetables and used less medication than prescribed due to cost.

Our results add to the evidence about how two SDoHs, educational attainment and household income, are related to SRs. Within all racial/ethnic groups, we found that high school graduates were more likely than college graduates to be in a low income group, as well as less likely to believe that their health habits and lifestyle could have a large effect on their health. In turn, those in the low income group were more likely than those with higher incomes to feel financial worry and less likely to have sufficient financial resources to eat a healthy diet and use medications as prescribed. These findings are consistent with previous research that shows lower levels of financial strain among individuals with higher levels of education [29] and that, compared to those with low educational attainment, individuals with high educational attainment are more likely to have financial resources to buy healthy foods and to access appropriate health care [30,31]. Consistent with previous research, we found that differences in the prevalence of SRs between those at lower and higher levels of educational attainment and income were wider within racial/ethnic groups than across racial/ethnic groups among those in the same education and income levels [32–38]. At a societal level, our findings support the importance of remediating underlying racial/ethnic group inequalities in educational attainment in order to reduce racial/ethnic disparities in social and financial risks, and ultimately, in health and well-being.

At the health plan level, these results suggest that more routine capture of educational attainment and measures of financial strain in the electronic health record system could help healthcare providers better understand social factors that may affect their patients’ self-care practices, healthcare utilization, and health outcomes and enable them to make more patient-centered recommendations. At the population health level, this information could facilitate identification of subgroups of patients who might be at higher priority for assessment of social/financial need and referral to internal health plan medical financial assistance, food assistance programs, and community-based organizations that can help with non-medical related social needs. Population health management and quality departments would be able to incorporate these factors as risk-adjustors in the ongoing measurement of racial/ethnic disparities in health outcomes and health care utilization. Finally, while health systems, alone, are not going to be able to remediate racial/ethnic disparities in educational attainment and income associated with structural racism, having this information about their patients will better prepare health systems for the necessary intersectoral collaborations (e.g. social services sector, education sector, housing sector, etc.) required to reduce racial/ethnic disparities in health and healthcare.

Overall, we found that among adults ages 35 to 64, over one-third of those in the lower income category and approximately one-fourth of those in the higher income category were very worried about their financial situation. Financial strain, whether based on current or anticipated financial situation, may lead to behavioral and psychosocial risks for poor health and reduced use of discretionary health care. Yet, many of these adults may not meet eligibility for government or community safety net programs to help with medical, dental, food, and other types of expenses. This suggests that in addition to having pathways for internal and community referrals for those who are eligible for safety net programs, patient navigators and social workers in inpatient and outpatient settings may face a more time-consuming and potentially more difficult task of helping vulnerable adults figure out how to make the best use of the resources they have. Healthcare providers, pharmacists, and administrators may need to explore creative ways to reduce a patient’s medication costs or spread payment out over time when patients indicate that they are struggling to cover medical costs along with other basic necessities. Most importantly, our results underscore that many adults who don’t qualify for Medicaid or other governmental programs for very low income families are or feel financially vulnerable. It is important for health care providers to keep this in mind before making universal recommendations that might be difficult for financially-challenged individuals to follow. For example, instead of only recommending that patients increase consumption of fresh fruits and vegetables, providers can mention the health benefits of alternatives such as eating frozen or canned fruits and vegetables. Healthcare providers can also make greater use of virtual (telephone, video) visits instead of an office visit, which are often lower cost, and can offer alternatives to prescription medications that might be available in a cheaper generic form or that might be helpful but not necessary. It is also important for health care providers to assess whether cost is a barrier when patients often miss or cancel office visit appointments, are not using medications as prescribed, or are not following dietary recommendations so that the process of finding solutions can begin.

Our finding that across all race/ethnic groups, younger adults in the lower and higher income categories were approximately twice as likely as older adults to be very worried about their financial situation also suggests household income may not adequately represent an individual’s actual and perceived financial circumstances when used for health services research or health plan means testing for subsidy programs. This is in line with a growing body of research that is identifying limitations to using traditional measures of socioeconomic status, such as income and education, when trying to describe the socioeconomic context of an individual’s health, health-related behaviors, and healthcare utilization. For example, some researchers are recommending that information about household or personal income be supplemented with other indicators of an individual’s financial and living situation, including current and anticipated demands on that income and different sources of material and psychological financial hardship and financial stress [39].

We found that feeling very worried about one’s financial situation was significantly associated with increased risk of chronic high stress. Within racial/ethnic groups, though, older adults were also less likely than younger adults to report being very worried about their financial situation. The results of our multivariable models suggest that chronic high stress was significantly associated with being very worried about one’s financial situation, while income and education were not; accordingly, this lower prevalence of financial worry may partially explain the lower prevalence of chronic stress reported by older adults. Among Whites, Blacks, and Latinos, the lower reported exposure to harassment or discrimination among the older adults may also contribute to the lower prevalence of chronic stress reported among adults in the younger age group.

To our knowledge, this study is among the first to investigate differences in SDoHs and SRs between Filipino-American and Chinese-American adults. Asian-Americans are the fast growing segment of the U.S. population, and Chinese-Americans and Filipino-Americans are the first and third largest Asian ethnic groups in the U.S., respectively, with the largest concentrations of both ethnic groups found in the state of California [40]. Despite widespread recognition that these ethnic groups are culturally very different, there has been very little research into how they differ on social factors that may affect their health and access to healthcare. We found that while both groups had higher percentages of adults with college degrees, higher percentages of Filipinos had no post-secondary education, and in the older group, higher percentages had not completed high school. In both age groups, Filipinos had higher percentages of adults in the lower income group, lower percentages in the middle and higher income groups, and higher percentages who were very worried about their financial situation, making them appear more similar to the results observed in Blacks and Latinos than in Chinese and Whites. Additionally, the percentages of Filipinos who believed that their health habits/lifestyle and emotional troubles could have a large effect on their health were much lower than Chinese and all of the other racial/ethnic groups. Filipinos and Chinese were similar in having high percentages of adults who were married and low prevalence of chronic stress and cost-related under-use of medications and fruit and vegetable consumption despite the differences between the two groups in income and financial stress. Differences in SDoH and SR profiles across these ethnic groups may contribute to the higher prevalence of obesity and chronic health problems that have been observed in ethnic Filipino-American adults compared to Chinese-American adults [41–50].

A strength of this study was its large, racially/ethnically diverse sample that enabled us to estimate and compare prevalence of SDoHs and SRs separately for middle-aged and older adults. The study sample also enabled us to identify several significant differences between Filipino-American and Chinese-American adults that would have been masked if our analysis had used an aggregated Asian racial group. However, our study had some limitations. The survey had a relatively low response rate for middle-aged adults and potential response bias (lower response rates for Blacks, Latinos, and Filipinos than Whites and Chinese and likely for people with low vs. high educational attainment). However, the response rate is actually higher than those of many random-digit-dial health surveys that have been the basis for previously published research on this topic. Another limitation is that the household income variable used to define low and high income did not adjust for number of people contributing to that income or number of people supported by that income. As such, the relationship of educational attainment with income was more tenuous than if the low and higher income categories had been derived from personal income. The study was based on data from adults with health insurance who receive care through a single health plan in Northern California and was restricted to people who could complete the survey in English and who had a usable mailing address, which limits generalizability to the general population. Finally, the study sample was not sufficient to take the next step of examining the association of these SRs with health and well-being within and across racial/ethnic groups and the extent to which they help explain racial/ethnic disparities that have been observed in previous research. However, there is evidence from previous studies suggesting a positive association of health status with education [30,51–54] and income [55–57] and a negative association of health status with financial strain [39,58,59]. Future research should focus on the pathway through which SRs influence health and healthcare use.

## Conclusions

In this non-“safety net” health plan population, we found several instances of racial/ethnic differences in social risks, with Blacks, Latinos, and Filipinos having a higher prevalence of social risks than White and Chinese adults. We also found differences in the prevalence of social risks between middle-aged and older adults. Some of these differences seem to be mediated by educational attainment or income. Differences in the prevalence of social risks by levels of education and income were wider within racial/ethnic groups than across racial/ethnic groups within the same levels of education and income, suggesting that these factors should be used as risk adjustors when studying and reporting on racial/ethnic differences in health and healthcare use. These results support the assessment and EHR documentation of SDOHs and social risks and use of this information to understand and develop population health management strategies to address the factors that put people "at risk of risks" [60] that contribute to racial/ethnic disparities in health and healthcare use.

## Supporting information

S1 Appendix Survey items used to create study variables. (DOCX)
