# Supplementary material for: Racial-ethnic differences in prevalence of social determinants of health and social risks among middle-aged and older adults in a Northern California health plan
Source: PLoS One. 2020 Nov 4;15(11):e0240822. doi: 10.1371/journal.pone.0240822 (PMC7641349; doi:10.1371/journal.pone.0240822)
Supplement: S1 Appendix — (DOCX) [file pone.0240822.s001.docx]

**S1 Appendix. Survey items used to create study variables**

**Social Determinants:**

**Education:**

What is the highest level of school you completed? *(Check only* ONE *answer)*

| □ 8th grade or less *(primary or middle school)* | | □ Some college *(no degree)* | |
| --- | --- | --- | --- |
| □ 9th - 11th grade *(some high school)* | | □ Associate’s Degree *(e.g., AA, AS)* | |
| □ 12th grade *(high school graduate or G.E.D.)* | | □ Bachelor’s Degree *(e.g., BA)*, teaching credential | |
| □ Technical/trade school certificate | | □ Graduate or professional degree *(e.g., MA, MD)* | |

**Income:**

Which of the following best describes your total household (family) income from all sources in [last year], before taxes?

| □ Under $15,000 | □ $35,001 - $50,000 | □ $80,001 - $100,000 |
| --- | --- | --- |
| □ $15,000 - $25,000 | □ $50,001 - $65,000 | □ $100,001 - $150,000 |
| □ $25,001 - $35,000 | □ $65,001 - $80,000 | □ More than $150,000 |

**Marital status:**

Are you currently: □ Married □ In a committed relationship □ Widowed □ Single, divorced, or separated

**Social Risks:**

**Financial worry and Harassment/Discrimination**:

During the past 12 months, did any of these situations occur?

□ You worried a great deal about your or your family’s financial security

□ You felt harassed or discriminated against

**Chronic high stress:**

During the past 12 months, how often have you felt very stressed, tense or anxious?

□ Never □ A little of the time □ Some of the time □ Much of the time □ Most of the time

**Reduced use of medications and fruit/vegetable consumption due to cost:**

During the past 12 months, did you:

a. Start to take a medicine in smaller doses or less frequently than prescribed, or decide

not to fill a prescription because of the cost? □ Yes □ No

b. Eat less fruit and vegetables than you wanted to because of the cost? □ Yes □ No

**Health-related beliefs:**

How much do you think habits/lifestyle (such as exercise, what you eat, and your weight) can affect your health? □ Not at all □ A little bit □ Moderately □ Quite a bit □ Extremely

How much do you think stress and emotional troubles (such as depression or anxiety) can affect your health?

□ Not at all □ A little bit □ Moderately □ Quite a bit □ Extremely
